# Influence of cold atmospheric plasma on dental implant materials — an in vitro analysis

**DOI:** 10.1007/s00784-021-04277-w

**Published:** 2021-12-15

**Authors:** Gunar Wagner, Benedikt Eggers, Dirk Duddeck, Franz-Josef Kramer, Christoph Bourauel, Søren Jepsen, James Deschner, Marjan Nokhbehsaim

**Affiliations:** 1 Department of Periodontology, Operative and Preventive Dentistry, Center of Dento-Maxilo-Facial Medicine, University of Bonn, Welschnonnenstr. 17, 53111 Bonn, Germany; 2 Department of Oral Surgery, Center of Dento-Maxillo-Facial Medicine, University of Bonn, 53111 Bonn, Germany; 3 Department of Prosthodontics, Geriatric Dentistry and Craniomandibular Disorders, University Charité Berlin, 14197 Berlin, Germany; 4 Research Department, CleanImplant Foundation, 10117 Berlin, Germany; 5 Department of Cranio-Maxillofacial Surgery, Center of Dento-Maxillo-Facial Medicine, University of Bonn, 53111 Bonn, Germany; 6 Department of Oral Technology, School of Dentistry, University of Bonn, 53111 Bonn, Germany; 7 Department of Periodontology and Operative Dentistry, University of Mainz, 55131 Mainz, Germany; 8 Section of Experimental Dento-Maxillo-Facial Medicine, Center of Dento-Maxillo-Facial Medicine, University of Bonn, 53111 Bonn, Germany

**Keywords:** Cold atmospheric plasma, Dental implants, Bone remodeling, Periimplantitis

## Abstract

**Background and objectives:**

Alterations in the microenvironment of implant surfaces could influence the cellular crosstalk and adhesion patterns of dental implant materials. Cold plasma has been described to have an influence on cells, tissues, and biomaterials. Hence, the mechanisms of osseointegration may be altered by non-thermal plasma treatment depending on different chemical compositions and surface coatings of the biomaterial. The aim of the present study is to investigate the influence of cold atmospheric plasma (CAP) treatment on implant surfaces and its biological and physicochemical side effects.

**Materials and methods:**

Dental implant discs from titanium and zirconia with different surface modifications were treated with CAP at various durations. Cell behavior and adhesion patterns of human gingival fibroblast (HGF-1) and osteoblast-like cells (MG-63) were examined using scanning electron microscopy and fluorescence microscopy. Surface chemical characterization was analyzed using energy-dispersive X-ray spectroscopy (EDS). Quantitative analysis of cell adhesion, proliferation, and extracellular matrix formation was conducted including real-time PCR.

**Results:**

CAP did not affect the elemental composition of different dental implant materials. Additionally, markers for cell proliferation, extracellular matrix formation, and cell adhesion were differently regulated depending on the application time of CAP treatment in MG-63 cells and gingival fibroblasts.

**Conclusions:**

CAP application is beneficial for dental implant materials to allow for faster proliferation and adhesion of cells from the surrounding tissue on both titanium and zirconia implant surfaces with different surface properties.

**Clinical relevance:**

The healing capacity provided through CAP treatment could enhance osseointegration of dental implants and has the potential to serve as an effective treatment option in periimplantitis therapy.

## Introduction

The main goal in modern dental implantology is to maintain biological stable conditions and prevent peri-implant diseases despite a high microbial load in the oral cavity and increased mechanical stress. The process of osseointegration is determined by a cascade of cellular and molecular mechanisms involving the recruitment of different cell types and levels of morphological differentiation as well as inflammatory conditions in conjunction with matrix proteins and growth factors [1]. Improvements for a precise interaction between the biomaterial and the cells of the target tissue could be achieved by considering the influence of the biomaterial’s surface properties on the cell–matrix crosstalk. Histological and biomechanical evidence strongly suggests that different surface alterations could manipulate soft and hard tissue integration and therefore may influence healing and anchorage of the dental implant [2, 3]. In this context, different surface structures and nanotechnologies for coating of dental implants have been developed and today, there is a broad range of them used in combination with various implant designs and materials on the market [4–6]. Systematic reviews highlight the different coatings on titanium surfaces to achieve better osseointegration and faster loading in clinical conditions [7, 8], such as calcium phosphate [9] or titanium dioxide or nanoparticles [10, 11]. In addition, in vitro studies indicate that zirconium dioxide (ZrO_2_) implants may promote osseointegration [12]. Nevertheless, peri-implant diseases are a growing challenge, but the pathogenesis is dissimilar to periodontitis showing distinct patterns of biofilm formation with diversity in corrosion/tribocorrosion for implant materials. Deregulation of inflammation triggered by changes of the microbiome and immune modulation of the individual can lead to severe tissue destruction [13, 14].

**Fig. 1:**
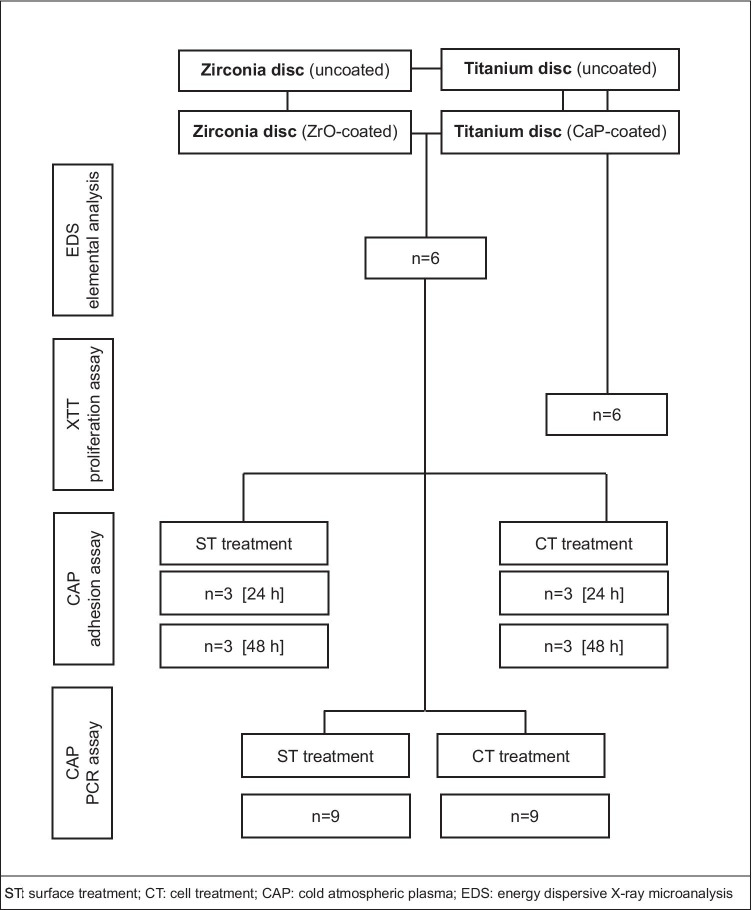
The design of the study with the sample size for each experiment

Decontamination techniques should promote optimal bacterial reduction without changing the topographical characteristics of dental implants favoring cellular recolonization and consequent re-osseointegration. Regarding the treatment of peri-implant diseases, it should be considered that different surface properties of zirconia and titanium must be considered. Despite the removal of bacterial pathogens, the elimination of organic nitrogen compounds represents a key factor in those efforts to restore and heal previously infected areas [15]. It could be revealed that biological reintegration of dental implants consistently occurred at sites where exposed surfaces were replaced with a pristine component [16]. Various innovative strategies using laser, photodynamic therapy, and ozone (O_3_) as well as cold atmospheric plasma (CAP) treatment have emerged to provide a stable debridement procedure that seeks to meet all necessary criteria for periodontal healing [17–20]. Cold atmospheric plasma is a highly reactive gas that has been described to generate a variety of reactive species (ozone, reactive oxygen, and nitrogen species) with antimicrobial effects [21]. Although the precise mode of action is still under investigation, similar efficacy to the effect of 0.2% chlorhexidine digluconat could be observed in bacterial biofilms [22]. Argon-based atmospheric plasma affects the initial adhesion of bone marrow cells and stimulates bioactivity on titanium implant surfaces [23]. CAP operates at room temperature utilizing portable equipment and represents a promising technology to achieve early osseointegration of biocompatible implant surfaces. Hence, it is a promising and affordable option to use during surgery immediately prior to implant placement or during periimplantitis treatment. In previous studies, we have demonstrated a stimulating effect of ambient air CAP on human periodontal cells, keratinocytes, fibroblasts, cementoblasts, and osteoblast-like cells [24–26]. Since it remains unclear to what extent the application of ambient CAP has an impact on cellular homeostasis on implant surfaces, we seek to determine the precise mechanism of CAP treatment on cells involved in osseointegration and soft tissue stabilization with possible material alterations on dental implants. The main aim was to provide a qualitative assessment for the treatment of titanium and zirconia materials using ambient CAP in vivo. Different surface modifications are examined to draw conclusions for clinical implications on the use of CAP either to promote osseointegration or as a possible tool for the treatment of peri-implant diseases (Fig. 1).

## Materials and methods

### Implant material samples

**Fig. 2:**
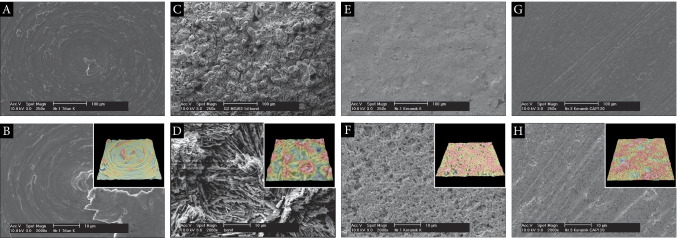
Scanning electron micrographs and 3D roughness reconstruction/heightmap calculation images (inserts). **A** Titanium grade IV specimen (× 250); **B** titanium grade IV (× 2000); machined surface (Sa: 0.76 μm); **C** coated titanium specimen (× 250); **D** coated titanium (× 2000); ultra-rough surface (Sa: 1.35 μm); **E** coated zirconia specimen (× 250); **F** coated zirconia (× 2000); medium rough surface (Sa: 0.43 μm); **G** milled zirconia specimen (× 250); **H** milled zirconia (× 2000); medium rough surface (Sa: 0.49 μm)

Commercially pure grade IV titanium discs with machined surface (ø 5.2 mm, thickness 1.5 mm/Sa: 0.76 μm; FairImplant™, Bönningstedt, Germany) were analyzed. Samples coated with a calcium-phosphate layer (BONIT®, ø 10 mm, thickness 2.0 mm/Sa: 1.35 μm) were used as comparison. DOT GmbH, Rostock, Germany, manufactured the coating layer. Zirconia discs comprising composite ceramic made of zirconium oxide and aluminum oxide (Ziraldent®, ø 5.2 mm, thickness 1.5 mm/Sa: 0.49 μm) were used in another group. Additionally, coated samples with a microporous implant surface made from proven zirconium oxide with equivalent dimensions were analyzed (Zircapore®, Sa: 0.43 μm). Metoxit AG, Switzerland, kindly provided zirconia specimens. Surface roughness was determined by the authors of this study using EDS (Fig. 2).

### CAP application

**Fig. 3:**
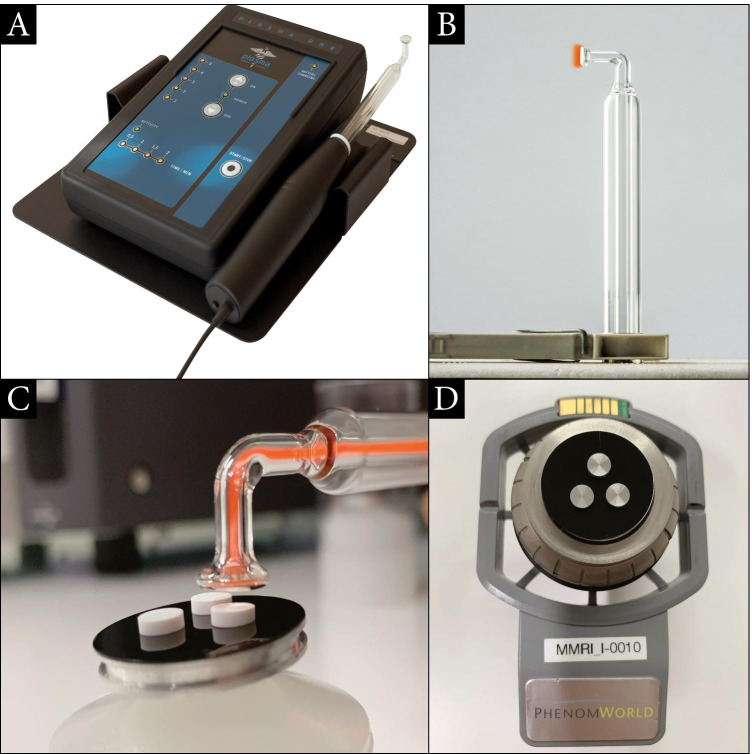
Cold atmospheric plasma (CAP) device experimental setting. **A** Energy control center with main unit, dental converter, patient probe, plasma device (Plasma ONE DENTAL); **B** glass instrument dental probe PS12 with transparent air chamber; **C** CAP application with ionization of atoms and molecules generating cold plasma. Zirconia specimens on sample holder; **D** EDS sample holder with titanium surface specimen (Phenom proX Scanning Electron Microscope)

Ambient air CAP was generated by a dielectric barrier discharge (Plasma ONE MEDICAL, Plasma MEDICAL SYSTEMS® GmbH, Nievern, Germany). Optimal time, intensity, and distance were selected after preliminary experiments (Kleineidam et al., 2019). The material surfaces of the specimens were exposed using CAP within a distance of 1 cm using the PS12 instrument probe at 18 kV. The time of application differed between 60 and 120 s of CAP treatment for different zirconia and titanium surfaces (Fig. 3).

### Cell culture and adherence experiment

Human osteoblast-like cells (MG-63) (ATCC, CRL-1427TM) (Sigma‐Aldrich, Taufkirchen, Germany) or Human Gingival Fibroblasts (HGF-1) (330703HPL; CLS Cell Lines Service GmbH, Eppelheim, Germany) were cultured in Dulbecco’s modified essential medium (DMEM, Invitrogen, Germany) supplemented with 10% fetal bovine serum (FBS, Invitrogen), 100 unit’s penicillin, and 100 μg/mL streptomycin (Invitrogen) at 37 °C in a humidified atmosphere of 5% CO_2_ and 95% humidity. For further studies, cells were seeded into 35 × 10 mm Petri dishes and cultured to 70% confluence. Cell culture medium was replaced every 2 days. For each experiment, FBS concentration was reduced to 1% 1 day prior to start. Different experimental groups were created to discriminate the effect of CAP between the cells and the surface properties. The first group received surface treatment (ST) of the specimen prior to cell seeding. In the other group, CAP stimulation of the cell suspension took place prior to seeding on the untreated implant surfaces (CT). A group without any treatment served as a control. Cell adhesion took place for 24 h and 48 h post CAP treatment using a concentration of (10^4^ cells /100 μl). After the incubation process, all specimens were rinsed properly with phosphate-buffered saline (PBS) and further processed for scanning electron microscopy. A quantitative analysis was conducted. Cell counting was performed with the aid of the ImageJ software.

### Scanning electron microscopy

To investigate morphological changes of CAP treatment on different implant surfaces, scanning electron microscopy was performed. Furthermore, specific patterns of cell adherence were evaluated for different cell types. After cell incubation with different CAP treatment modalities (ST/CT), a fixation process was conducted and specimens were sputter coated with a thin layer of titanium in a sputter coating unit (Scancoat Six, HHV, Crawley, United Kingdom). Examinations took place by scanning electron microscopy (Philips XL-30 ESEM). High-resolution images with a magnification of up to 2000 × were obtained for in-depth analysis of cell behavior on different surfaces. Microscopic images were taken with magnifications of 50 × choosing a random area of interest to allow for further quantitative analysis (Fig. 2). The study was performed in triplicate for each cell line after 1 or 2 days of incubation on zirconia discs. Due to a sparse contrast of cells incubated on coated titanium surfaces, applying scanning electron microscopy was not effective for quantitative analysis. Additional experiments were performed using fluorescence microscopy for these specimens.

### Energy-dispersive X-ray microanalysis (EDS)

EDS was performed to analyze possible shifts in the elemental composition of different oral implant materials treated with CAP. The scientific workstation (Phenom proX Scanning Electron Microscope, Eindhoven, Netherlands) is equipped with a high-sensitivity backscattered electron (BSE) detector. The detector for the EDS and elemental analysis was a thermoelectrically cooled silicon drift detector (SDD) type, with a take-off angle (TOA) measuring 29° an active detector area of 25 mm^2^. Titanium and zirconia discs were mounted on the sample holder with carbon tabs without touching the sample surface and analyzed with a scanning electron microscope in a particle-free clean room environment (according to Class 100 US Federal Standard 209E, Class 5 DIN EN ISO 14644–1) to avoid artifacts from the ambient air. The BSE detector allows for magnification of up to 100.000 × with a resolution down to 15 nm. Within this study, material-contrast images from 500 × to a magnification of 10.000 × were obtained.

**Fig. 4:**
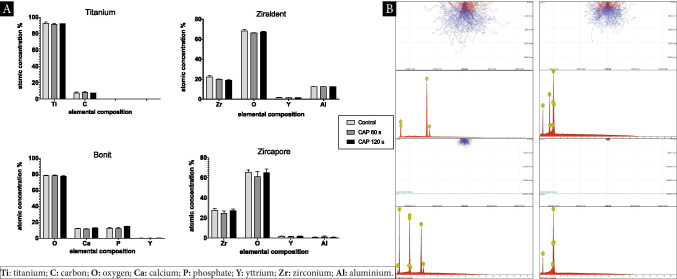
EDS elemental analysis using Phenom Element Identification software 3.8.4.0. **A** Quantitative EDS analysis for CAP application identifying shifts of the atomic concentration for implant materials. Influences on the elemental composition using application times of 60 s/120 s. **B** Monte-Carlo simulation for EDS spectra and electron profiles of different implant surfaces/blue dotted line indicating the coating layer of titanium (Bonit®) and superficial layer of zirconia (Zircapore®) specimens. All the statistical significance values were declared as *p* < 0.05

To analyze the exact chemical composition of each sample, the acceleration voltage and atomic number of the element most characteristic of the substance’s properties were adapted to investigate the outer layer (3 μm) of the sample’s surfaces. Calibration was done using a reference measurement by mapping Cu/Al (8.040 keV/1.486 keV). The working distance was 3 mm with the field of view (FOV) at 53.8 μm using a magnification of 5.000 × . Composition of the samples was assumed to be homogeneous. Microscopic analysis at three different detection points for each sample was done and EDS point analysis could be conducted. A reliable identification of elemental composition could be assumed with deconvolution measurements for X-ray quanta exceeding a minimum threshold (peak fit ≥ 0.99). The proportion of atomic concentration and weighted concentration of the elements before and after CAP treatment were measured and footprints of the treated area were statistically calculated. Analysis of the spectra and processing of the data was conducted using software (Phenom Element Identification 3.8.4.0; ThermoFisher Scientific, Eindhoven, Netherlands). Monte-Carlo simulation is used for calculating the profiles of the target surface topography, illustrating the interaction between X-ray signals and volume of the samples. Simulation was set up with an acceleration voltage of 15 keV using 10,000 electrons. Coated samples were calculated for layer thickness according to manufactures specification with 20 μm. All experiments, as well as the complete setup, as described above were performed at the Medical Materials Research Institute, Berlin, Germany. The laboratory is accredited according to DIN EN ISO/IEC 17,025 (Fig. 4).

### Analysis of gene expression

Twenty-four hours after CAP application, total RNA was extracted using an RNA extraction (RNeasy) kit (Qiagen, Hilden, Germany). NanoDrop ND-2000 spectrophotometer (Thermo Fisher Scientific, Wilmington, DE, USA) was used to measure the RNA concentration. A total of 1 μg of RNA was reverse transcribed into cDNA by use of iScript™ Select cDNA Synthesis Kit (Bio-Rad Laboratories, Munich, Germany) at 42 °C for 90 min followed by 85 °C for 5 min. One microliter of cDNA was amplified as a template in a 25 μl reaction mixture containing 12.5 μl SsoAdvanced™ Universal SYBR® Green Supermix (Bio-Rad), 2.5 μl of specific primers (0.5 μM each; predesigned QuantiTect Primer Assay, Qiagen), and 9 μl deionized water. The mixture was at first heated at 95 °C for 5 min, and then followed by 40 cycles with denaturation at 95 °C for 10 s and combined annealing/extension at 60 °C for 30 s. mRNA expression of Ki67, Proliferation-Cell-Nuclear-Antigen (PCNA), and Collagen-Typ1 alpha (COL1A1) was detected by real-time PCR using the iCycler iQ™ detection system (Bio-Rad). Glyceraldehyde 3-phosphate dehydrogenase (GAPDH) was used as an endogenous control. The data was analyzed by the comparative threshold cycle method.

### Analysis of cell proliferation

XTT (Cell Signaling Technology, Danvers, USA) kit was used to determine the cell viability on 96-well plates. CAP-treated groups (60 s/120 s), as well as untreated control of each cell type, were seeded on coated and non-coated titanium discs and incubated for 24 h. Next, XTT reaction solution was added to each group according to the manufacturer’s instructions. Measured specific absorbance (SA) was determined after 4 h of incubation with a microplate reader (Epoch™ Microplate Spectrophotometer, BioTek Instruments, Winooski, USA). Optical density (OD) was determined at 450 nm (reference wavelength 690 nm). For quantifications, the background levels of media without cultured cells were subtracted. Additionally, cell numbers of each group were determined by using an EVE™ (NanoEnTek Inc., Seoul, Korea) automatic cell counter 24 h after both CAP application periods (60 h/120 s) of MG-63 cells as compared to untreated control.

### Fluorescence microscopy

For visualization and quantitative analysis of titanium samples, we performed fluorescence staining of the cells at different time points. Cell suspensions of each cell type were prepared and stimulated as described above on both non-coated or pre-coated titanium samples for 24 h and 48 h. Cell adhesion was analyzed with a Phalloidin/DAPI staining after fixation with 4% paraformaldehyde (Merck, Darmstadt, Germany) for 10 min and permeabilization with 0.05% Triton X-100 (Merck) for 5 min in PBS. One hundred micromolar phalloidin (Merck) was used for 40 min in order to label the actin filaments. After washing, 1 µg/ml DAPI (Merck) was applied for 3 min to label DNA. Stained cells were embedded with Mowiol (SigmaAldrich) and evaluated with the ZOE™ Fluorescent Cell Imager (Bio-Rad). The study was performed in triplicates and repeated two times for each cell. Six different areas of interest (AOI) were randomly assigned for calculation. In total, 72 AOI were analyzed for cell attachment and pooled probes were quantitatively analyzed. For further quantitative measuring, nucleus staining was allowed for cell counting.

### Statistical analysis

The design of the study with the sample size for each experiment is displayed (Fig. 1). For statistics, the GraphPad Prism9 Software (GraphPad Software, San Diego, USA) was used. All experiments were performed in triplicate and reproduced at least twice. For statistical comparisons between groups, the Kruskal Wallis test followed by the post hoc Dunnett’s tests with corrected *p*-values were used for multiple comparisons. Differences between groups were considered significant at *p* < 0.05. All data are presented as mean with standard error of the mean (SEM). EDS data were analyzed using two-way ANOVA followed by the post hoc Tukey’s multiple comparison test using corrected *p*-values.

## Results

### EDS analysis for surface elemental composition

To investigate the influence of CAP on different implant materials and surface modifications regarding their topographical and physiochemical properties, EDS was performed. We speculated that exposure time could possibly have led to a shift in elemental composition on the atomic level. The EDS survey analysis of pure titanium implant surfaces revealed a large amount of titanium 92.5% ± 2.41 and a fraction of carbon 7.4% ± 2.41. Application of CAP showed no significant alterations in percentage composition irrespective of the exposure time (120 s). Calcium phosphate coated titanium specimens (BONIT®) showed to have a large amount of oxygen 78.1% ± 1.48 and a minor proportion of calcium 11.9% ± 1.06 and phosphate 9.8% ± 0.34. Only traces of yttrium could be detected (Table 1). EDS was not able to detect any signals of titanium within coated specimens since the coating layer of 20 μm was not penetrated by X-ray signals, which only reached a detection depth of 5 μm demonstrated through Monte-Carlo simulation (Fig. 4B). Zirconia specimens (Ziraldent®) comprise a large amount of oxygen 68.1% ± 2.98 and approximately one-quarter of zirconia 21.4% ± 3.59 with a minor proportion of aluminum 12.3% ± 0.32 as well as small amounts of yttrium 1.5% ± 0.17. This chemical composition could not be altered by CAP treatment irrespective of the exposure time. High-resolution spectrum analysis confirmed comparable levels of oxygen, zirconia, and yttrium in those specimens (Zircapore®). Although some shifts in carbon composition in the coated zirconia group could be observed, the standard deviation in the test objects was high and no significant results could be drawn regarding the influence of CAP.

**Table 1:** EDS elemental analysis for both untreated and plasma treatment (+ CAP) surfaces (mean SEM)

surface material	Ti [mass%]	C [mass%]	O [mass%]	Ca [mass%]	P [mass%]	Y [mass%]	Zr [mass%]	Al [mass%]
Titanium	92.5 ± 1.39	7.4 ± 1.39	-	-	-	-	-	-
Titanium + CAP	91.5 ± 0.72	8.4 ± 0.72	-	-	-	-	-	-
Bonit	-	-	78.1 ± 0.60	11.9 ± 0.43	9.8 ± 0.14	**	-	-
Bonit + CAP	-	-	78.4 ± 0.44	11.7 ± 0.33	9.6 ± 0.13	**	-	-
Ziraldent	-	-	68.1 ± 1.12	-	-	1.5 ± 0.06	21.4 ± 1.36	12.3 ± 0.14
Ziraldent + CAP	-	-	67.4 ± 0.85	-	-	1.5 ± 0.03	20.7 ± 0.80	11.5 ± 1.06
Zircapore	-	n.s	65.4 ± 2.48	-	-	1.9 ± 0.09	27.8 ± 1.37	0.99 ± 0.09
Zircapore + CAP	-	n.s	61.1 ± 5.35	-	-	1.6 ± 0.15	24.8 ± 2.05	1.3 ± 0.66

Tr titanium; C: carbon: O: oxygen; Ca: calcium: P: phosphate: Y: yttrium: Zr: zirconium: AI: aluminium. **Only trace amounts were present; n.s: data not significant

Aluminum was significantly reduced in comparison with non-coated specimens 0.99% ± 0.20. Although the penetration depth using EDS was not able to reach the underlying surface of coated zirconia specimens, as shown for coated titanium objects using Monte-Carlo simulation, the main fractions remain stable for the basic elemental composition with no significant changes caused by CAP treatment.

### XTT analysis for cell proliferation

**Fig. 5:**
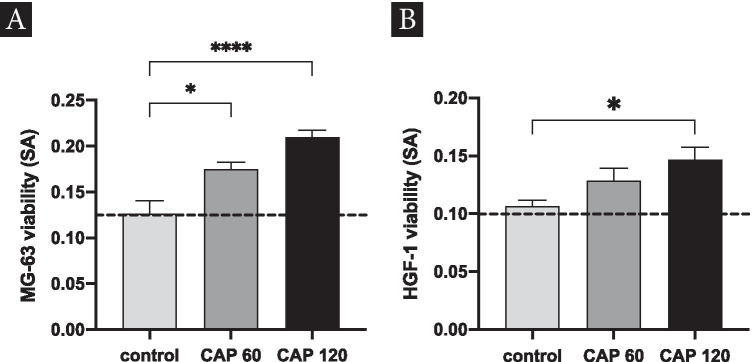
Determination of cell proliferation by XTT assay applying CAP at various durations. **A** Proliferation of MG-63 cells after CAP application with 60 s/120 s (intensity: 18 kV); **B** proliferation of HGF-1 cells after CAP application with 60 /120 s (plasma device intensity: 18 kV); columns and error bars represent the mean and SEM of measured specific absorbance (SA)/(OD blanked 450–690 nm). Mean ± SEM (*n* = 6); *significant (*p* < 0.05); **** significant (*p* < 0.0001) difference between groups

Changes in cell proliferation of osteoblast-like cells (MG-63), as well as human gingival fibroblast (HGF-1), were investigated using different application periods of CAP. Using an XTT assay, a significant increase in cell proliferation for MG-63 cells after 60 s of CAP treatment could be revealed (*p* < 0.05). Furthermore, an application time of 120 s caused a 1.6-fold increase of cell proliferation (Fig. 5A). When analyzing gingival fibroblasts, a constant increase in cell proliferation could be observed in both treated CAP groups. The application time of 120 s led to a 1.4-fold increase compared to the control yielding significant results (Fig. 5B).

### Adhesion assay

**Fig. 6:**
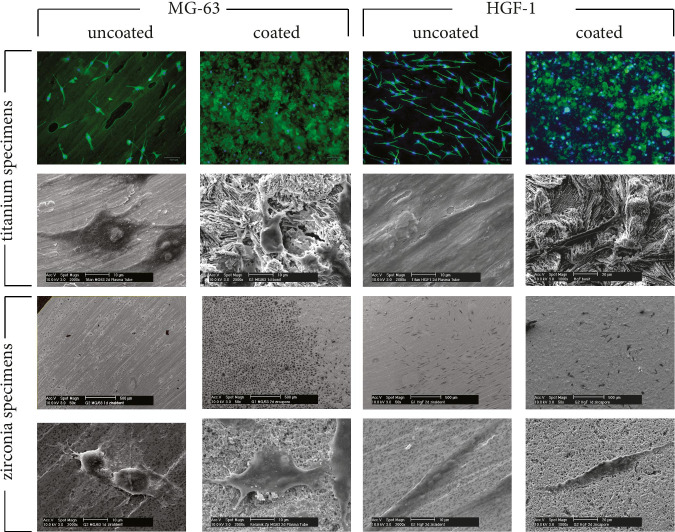
Scanning electron microscopy/fluorescence microscopy detecting adhesion assay on titanium/zirconia specimens. Different cell types (MG-63) and (HGF-1) (columns); coated/uncoated implant surface materials (rows). Detailed images showing cell adhesion of cells using high magnification (× 2000). Fluorescence microscopy revealing staining of cell nuclei with DAPI (blue) and the cytoskeleton using phalloidin staining (green)

The influences of CAP treatment on the adherence capability for oral cells regarding different dental implant materials were evaluated. In-depth analyses with high magnifications using scanning electron microscopy could demonstrate sufficient cell attachment on the implant materials investigated. Different patterns of adhesion between zirconia specimens coated (Zircapore®) and non-coated (Ziraldent®) were investigated. Fluorescence microscopy was used to detect cell adhesion on coated (Bonit®) and non-coated (titanium grade IV) titanium specimens. Staining of cell nuclei with DAPI allowed for high-contrast display of MG-63 cells and HGF-1 even on coated titanium surfaces with a high background noise (Fig. 6).

**Fig. 7:**
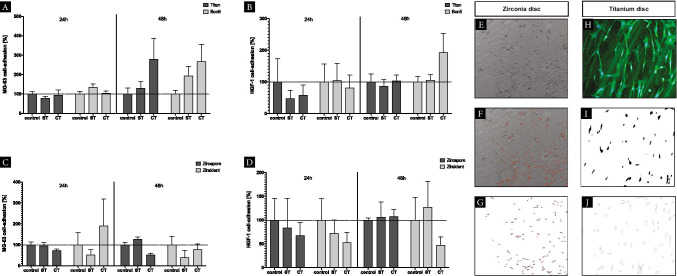
CAP adhesion assay. **A–D** Adjusted percentage of cell adhesion after (24 h/48 h) of different CAP treatment modalities. Untreated specimens/cells served as control, surface treatment (ST), cell treatment (CT); columns and error bars represent the mean ± SEM (*n* = 3). **E/H** Cell quantification using ImageJ Software, microscopic detection of cell colony within the area of interest (AI) (× 50). **F/I** Image transformation for cell detection using 8-bit resolution (Process: Binary: Make Binary) with cell separation **G/J** automatic cell counting (Menu: Analyse Particles) narrowing particle size (Size: 25-Infinity/Circularity: 0.00–1.00)

Within an observation period of 24 h, MG-63 cell adhesion on titanium grade IV implant surfaces that had received stimulation with CAP prior to cell seeding (ST) was not altered. CAP stimulation of the cell suspension prior to seeding (CT) did not result in advanced cell adhesion. CAP treatment of coated titanium surfaces with a calcium-phosphate layer (Bonit®) resulted in 1.3-fold increase of cell adhesion for MG-63 cells after an observation period of 24 h (ST). In contrast, the pretreatment of cell suspension prior to seeding showed no significant effects (CT). After 48 h of incubation, a 2.8-fold increase on cell adhesion could be observed for MG-63 cells with pretreatment of the cell suspension whereas the surface treatment with CAP resulted in minor increases of 1.3-fold compared to the control. Similar observations could be confirmed with coated surfaces (Fig. 7A).

HGF-1 adhesion on titanium grade IV was reduced after surface stimulation (ST) or treatment of the cell suspension (CT) by 0.5-fold within 2 h. If using longer observation periods of up to 48 h, no significant influences of CAP treatment regarding HGF-1 adhesion on titanium grade IV could be observed compared to the control. Same was evident regarding cell adhesion of HGF-1 on CAP-treated titanium specimens with coating (ST) after 24 h with a slight downregulation in (CT). Interestingly, given an observation period of 48 h, a significant 1.9-fold increase in cell adhesion for HGF-1 was observed (Fig. 7B). The evaluation of cell adhesion on zirconia resulted in divergent patterns compared to titanium specimens. Uncoated zirconia surfaces (Ziraldent®) showed less cell adhesion of MG-63 cells when pretreated (ST) with CAP after 24 h/48 h (0.5/0.4-fold). An increase of up to 1.9-fold of cell adhesion was measured after 24 h with cell treatment (CT) although data showed a high variation of responses. Coated specimens revealed no changes after 24 h and a slight increase after 48 h compared to the control receiving surface treatment (ST). In contrast, cell treatment with CAP (CT) showed a decrease in adhesion of MG-63 cells (Fig. 7C). Constant decrease in cell adhesion was observed for HGF-1 compared to the control on zirconia specimens in both coated and uncoated surfaces within 24 h independent from the treatment method. Coated specimens (Zircapore®) revealed a decrease of cell adhesion (0.6/1.7-fold). Comparable patterns of decreased cell adhesion for HGF-1 were observed within 48 h for uncoated specimens except for surface treatment (ST) where an upregulation (1.2-fold) was observed. For coated specimens (Zircapore®), a slight increase compared to the control (0.6/0.7-fold) after 48 h was evident. The variance (SEM) was high in this experiment. Adjusted *p*-values > 0.99 show no significance (Fig. 7D).

### Influence of CAP on MG-63 gene expression

**Fig. 8:**
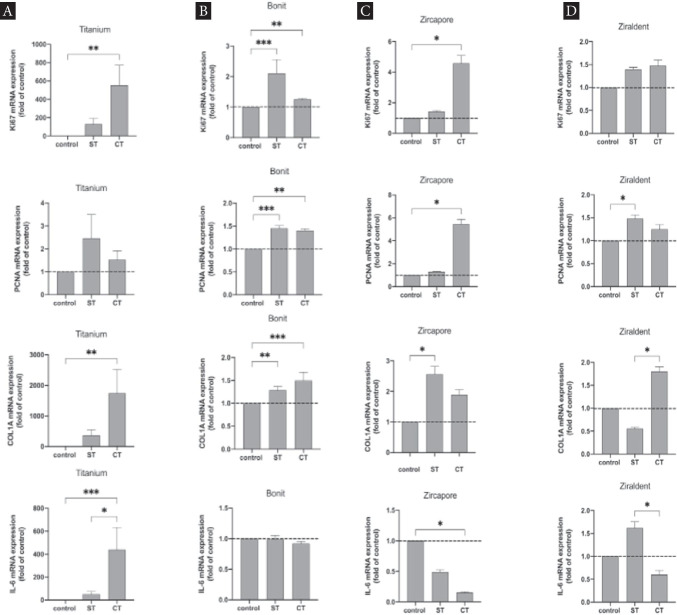
CAP influence on mRNA expression of osteoblast-like cells (MG-63) using different implant surfaces. **A** Uncoated titanium specimen. **B** Coated titanium specimen (Bonit®), untreated specimens/cells served as control, surface treatment (ST), cell treatment (CT).** C** Coated ceramic specimen (Zircapore®) untreated specimens/cells served as control, surface treatment (ST), cell treatment (CT) (plasma device intensity: 18 kV). **D** Uncoated ceramic specimen (Ziraldent®) (plasma device intensity: 18 kV); columns and error bars represent the mean ± SEM (*n* = 9); statistical difference between groups: *p < 0.05; **p < 0.005; ***p < 0.0001

When analyzing gene expression of MG-63 cells on uncoated titanium grade IV specimens, a significant increase of mRNA expression for Ki67, COL1A (*p* < 0.005), and IL-6 (*p* < 0.0001) was evident for CAP stimulation of the cell suspension prior to seeding (ST). PCNA was increased within both groups (ST, CT) although data were not significant (Fig. 8A). Analyzing the mRNA expression on coated titanium specimens (Bonit®), a significant upregulation was observed for implant surfaces that received stimulation with CAP prior to cell seeding (ST). Ki67 and PCNA were upregulated (*p* < 0.0001) as well as COL1A (*p* < 0.005). Additionally, a significant increase in mRNA expression was examined for treatment of cell suspension (CT) for these genes. The mRNA expression of IL-6 thus was not changed significantly (Fig. 8B). Analysis of coated zirconia specimens (Zircapore®) revealed upregulation of mRNA expression for Ki67 and PCNA comparing CT with the control (*p* < 0.05). COL1A was upregulated significantly after CAP stimulation of the surfaces (ST) whereas values for IL-6 were decreased and showed significant downregulation compared to the control (*p* < 0.05) (Fig. 8C). Uncoated zirconia specimens revealed an upregulation for Ki67 (not significant). PCNA was increased when comparing ST with the control (*p* < 0.05). A difference between surface treatment (ST) favoring cell suspension treatment (CT) was evident for COL1A (*p* < 0.05). The mRNA expression for IL-6 was upregulated when CAP treatment of the surface was performed (ST). In contrast, treatment of the cell suspension (CT) led to a significant downregulation compared to ST. Compared to the control, the expression of IL-6 was reduced (Fig. 8D).

## Discussion

The antimicrobial capacity of CAP using ambient air could be proven in past studies with varying efficacy regarding different bacterial species. Additionally, CAP was shown to be effective in eradicating biofilm activity on implant materials [27–30]. Strategies investigating the potential role of adjunctive treatment using argon cold plasma device during biofilm removal on titanium surfaces demonstrated enhanced regrowth of osteoblast cells. Whereas a combination of an optimized air polishing and argon plasma application could not prove enhanced osteoblast spreading on rough implant surfaces [31, 32]. Moreover, preclinical animal studies with a focus on argon plasma treatment revealed enhanced bone-to-implant contact (BIC) after functionalization of implant surfaces [33]. A recent meta-analysis came to conclusion that plasma treatment using argon may present an effective method for improving osseointegration [34]. Other investigations have also demonstrated enhanced osseointegration through ambient air-generated CAP for titanium surfaces and calcium-phosphate-coated implants [35]. The surface elemental chemistry was modified by this treatment resulting in a higher degree of exposure for adsorbed carbon species immediately after plasma treatment [36]. Interestingly, other studies investigating the influence of CAP on implant osseointegration revealed higher levels of carbon within their specimens (45.0% ± 5.1) compared to our observations and a decrease when plasma treatment was performed (38.0% ± 4.7). The fraction of titanium was significantly lower in this study using Ti-6Al-4 V bulk alloy implants. Increased levels of titanium (19.0% ± 3.9) and oxygen (43.0% ± 4.3) could be observed when plasma treatment was performed. Calcium-phosphate-coated implants showed lower values of oxygen (42.0% ± 5.2) compared to our data but comparable values for calcium and phosphate. The authors showed an increase of those values after CAP treatment [37]. Recently, the elemental composition and purity of dental implant surfaces of commercial zirconia implants have been investigated using non-destructive energy-dispersive X-ray spectroscopy (EDS) revealing comparable results to our investigation. The amount of aluminum within the specimens was similar to rough areas used for sampling of commercially available dental zirconia implants [38]. Monte-Carlo simulation was able to demonstrate that EDS analysis failed to detect the surface below the outside layer of titanium and zirconia specimens to analyze changes of the underlying elemental composition. It can be speculated that CAP, which leads to the ionization of atoms and molecules, also does not penetrate the surface layer within 20 μm. However, manipulation of the elemental chemistry of dental implants by plasma treatment could possibly lead to legislative implications affecting the European medical device regulation [MDR] effective from May 2021. Our data revealed no alterations in the basic elemental composition. Neither for titanium specimens nor for zirconia surfaces irrespective of different application times. CAP has shown to alter the surface energy and chemical topography due to the generation of high concentration of reactive species increasing the wettability and initial cellular interaction [39]. Additionally, plasma treatment can facilitate early osseointegration of any biocompatible implant surface regardless of its chemistry and topography change [40]. For a complete elemental and isotopic analysis of the implant body with high sensitivity and low detection limits down to the ultra-trace level (< 0.0001 mass%, equivalent to < 1 ppm), inductively coupled plasma mass spectrometry (ICP-MS) and inductively coupled plasma optical emission spectrometry (ICP-OES) are currently the methods of choice [41]. We could demonstrate that CAP treatment increases proliferation and viability of MG-63 cells and HGF-1 on titanium surfaces. Using application times of 60 to 120 s suggests that CAP could be beneficial to promote wound healing around dental implants as described earlier [26, 42]. Additionally, other cell types such as fibroblasts and epithelial cells, as well as keratinocytes, have been described to show higher levels of cell migration under the influence of cold plasma treatment [43–45]. Further in vitro and in vivo studies need to clarify the specific cellular context. Direct or indirect influences of CAP treatment must be differentiated since we could reveal different cellular patterns dependent on the application method. Adhesion assays were performed since the phases of cell-biomaterial interactions are crucial and destine to produce growth and differentiation factors to further establish innate biophysical properties of the implant surfaces [46]. Surface topography determines the cellular attachment of oral cells [47]. A variety of studies have explored the impact of roughness leading to improved techniques to refine surface conditions of abutments and fixtures [48–50]. Smooth titanium surfaces favor fibroblast attachment whereas rougher blasted surfaces promote osteoblast proliferation. Apical migration of junctional epithelium can be expected compared to grooved surfaces implicating that a certain threshold roughness (Ra = 0.22 µm) promotes a stable soft tissue seal. Initial attachment and spreading of human gingival fibroblasts are influenced by the surface texture of ceramic abutments [50]. We could not compare cell adhesion of MG-63 cells HGF-1 based on the surface layer because sizes of the specimens differed given the industrial manufacturing process. Thus, the number of cells that were used for the adhesion experiment was not equal in all experiments. Nevertheless, according to other studies, we could confirm that soft tissue sealing by fibroblast adhesion could be positively influenced by non-thermal plasma treatment [51, 52]. Likewise, the adhesion of MG-63 cells is positively influenced by CAP treatment on titanium surfaces given the opportunity of enhancing the osseointegration capacity. This early biological response of gingival cells needs to be reevaluated since we cannot outline possible detrimental influences by the saturation effect leading to rapid cell growth on the small zirconia discs. Furthermore, the effects of CAP on zirconia surfaces are not unambiguous and should be investigated using larger sample sizes and equal dimensions of the specimens. CAP treatment of MG-63 cells revealed different patterns of gene regulation affecting markers responsible for tissue remodeling of the extracelllular matrix (ECM). A significant upregulation of Ki67 and PCNA is observed when sample surfaces of cell suspensions are treated. This process of active cell proliferation due to CAP treatment could be confirmed [24]. The ossification process is promoted by osteoblasts through gene expression of COL1A [53, 54]. Treatment of cell suspensions with CAP leads to upregulation on all investigated implant surfaces implying promotion of osseointegration. Proinflammatory genes like IL-6 are regulated differently with irregular patterns of regulation comparing different titanium and zirconia specimens. Evaluation of mRNA levels was conducted 7 days after CAP application since the surface area of the specimens yielded only small quantities of RNA within 2 days of seeding. Thus, data of mRNA levels represent long-term effects of CAP treatment. Hence, it became obvious that CAP effects on gene expression are time dependent. Recent studies on the effect of CAP on apoptosis in MG-63 cells imply that CAP might help to enhance the healing of chronical hard tissue wound [24]. It remains unclear if CAP could be a useful tool in the treatment of mucositis or periimplantitis. Further studies would have to aim on observations focusing on the nano-level not only on the micron-level of implant surfaces to reveal possible shifts in terms of surface energy, wettability, and initial bone-implant contact in a time-dependent manner comparing ambient and argon plasma treatment alone or in conjunction.

## Conclusion

Regarding possible implications for CAP on medical implants we conclude that adjunctive treatment of titanium and zirconia surfaces could possibly enhance the healing capacity in difficult clinical situations with poor bone quality and quantity. Coating of implant surfaces may alter the effects of CAP on gene expression influencing the ECM. This therapeutic strategy could ultimately promote the osseointegration process of dental implants even for patients where bone metabolism is reduced.

## Limitations

The main limitation of this study is a small sample size increasing the margin of error. The adhesion assay using different implant materials could have gained more statistical power using specimens with equal dimensions in a larger quantity. Nevertheless, a trend could be observed on the effects of CAP treatment providing a scientific basis for an adequate sample size calculation to support further studies. It should also be noted that MG-63 osteoblast-like cells were used for the study. While these cells are commonly used as hard tissue cells, human osteoblasts better represent the hard tissue situation around implants [46, 48]. However, we focused on these cells because of their easier handling. Further studies on human osteoblasts from the jawbone are necessary before clinical application. To reveal clinical implications for soft tissue integration of dental implants treated with CAP, the use of periodontal ligament cells can be considered. Additionally, it should be kept in mind that for CAP treatment, a specific concentration cannot be defined, as would be possible with drug treatments. The only thing the dentist can adjust in CAP application is the treatment time, leading to time-dependent effects [49]. In the present study, we focused ourselves on a CAP treatment of 60 s and 120 s. It should also be kept in mind that implants have a cylindrical surface, which is only comparable to platelets to a limited extent. A treatment of this surface would therefore take significantly longer. Further treatment times and other surface conformations should therefore be the focus of future studies using standardized techniques. In the present study, we focused on analysis of mRNA expression of Ki67, PCNA, COL1a1, and IL6. Due to the number of other analyses, we have limited ourselves to these crucial markers for proliferation, extracellular matrix, and inflammation. The influence of CAP on mRNA expression of different cells, such as TGFβ, VEGF, IL8, APAF1, or CASP3, has been described in the literature [50, 51, 55]. Further studies should clarify how these and markers such as other cytokines and chemokines, matrix metalloproteinases, or apoptotic factors in cells on implant surfaces are influenced by CAP. As various other strategies for peri-implant therapy are also known, e.g., the application of CHX, the use of antibiotics, or the application of lasers or photodynamic therapy, a combination of CAP with one or more of these methods may present possible synergistic effects.
